# Hormone Replacement Therapy in Post-Menopause Hormone-Dependent Gynecological Cancer Patients: A Narrative Review

**DOI:** 10.3390/jcm13051443

**Published:** 2024-03-01

**Authors:** Paola Villa, Valentina Elisabetta Bounous, Inbal Dona Amar, Federica Bernardini, Margherita Giorgi, Daniela Attianese, Annamaria Ferrero, Marika D’Oria, Giovanni Scambia

**Affiliations:** 1 Department of Women and Child’s Health Sciences and Public Health, Fondazione Policlinico Universitario A. Gemelli IRCCS, 00168 Rome, Italy; paola.villa@policlinicogemelli.it (P.V.); amar.inbald@gmail.com (I.D.A.); federica.bernardini@guest.policlinicogemelli.it (F.B.);; 2 Gynecology and Obstetrics Unit, Mauriziano Umberto I Hospital, Department of Surgical Sciences, University of Turin, 10128 Turin, Italy; margherita.giorgi@unito.it (M.G.); d.attianese2807@gmail.com (D.A.); annamaria.ferrero@unito.it (A.F.); 3 Independent Researcher, 00186 Rome, Italy; marika.doria@outlook.it

**Keywords:** hormone replacement therapy, ovarian cancer, cervical adenocarcinoma, endometrial cancer, menopause

## Abstract

Background. Advances in the treatment of gynecological cancer have led to improvements in survival but also an increase in menopausal symptoms, especially in young women with premature iatrogenic menopause. Methods. A narrative review was performed to clarify the possibility of prescribing hormone replacement therapy (HRT) after hormone-dependent gynecological cancers (ovarian cancer [OC], cervical adenocarcinoma [AC], and endometrial cancer [EC]). Results. HRT can be prescribed to patients with early-stage, grade I–II OC who experience bothersome menopausal symptoms non-responsive to alternative non-hormone therapy after optimal surgery. Caution should be exercised in administering HRT after serous borderline tumors and endometrioid OC, and HRT is not recommended in low-grade serous OC. HRT is not contraindicated in AC survivors. After surgery for EC, HRT can be prescribed in women with early-stage low-grade EC. There is not enough data to give indications to patients with advanced EC. Conclusions. HRT can be discussed with patients, evaluating the risks and benefits of hormone-dependent gynecological cancer. Counseling should be performed by gynecologic oncologists experienced in the management of these patients.

## 1. Introduction

Hormone-dependent gynecological cancers (ovarian cancer [OC], cervical adenocarcinoma [AC], and endometrial cancer [EC]) are most frequently diagnosed among postmenopausal women; however, one-fourth of them occur in premenopausal women. The therapeutic improvements for these cancers have led to an increase in survival rates, with a consequently higher number of long-term survivors. Nevertheless, the treatment itself can lead to iatrogenic menopause in young women, with symptoms that can negatively impact quality of life (QoL). Women treated for hormone-dependent gynecological cancers must face the consequences of estrogen deficiency resulting from the surgical resection of ovaries, adjuvant postoperative irradiation, concomitant chemotherapy, or simply due to natural aging after menopause. The menopausal symptoms most commonly reported by young women with iatrogenic menopause include vasomotor symptoms, followed by those associated with the genitourinary syndrome of menopause (GSM), bone loss, and cognitive impairment [1].

In a multicenter study investigating the main menopausal symptoms in 166 OC survivors, 36% of women had surgical menopause, and half of the patients reported vasomotor symptoms, while two-thirds of OC women reported a decrease in libido [2].

These symptoms can have a negative impact on the sexual functions of young women with a history of hormone-dependent gynecological cancer. Sexuality is an essential component of female health, and women with cancer try to preserve this aspect of their well-being, even if they rarely seek medical assistance [3]. 

Surgical induction of early menopause enhances the risk of ischemic stroke, doubles the lifetime risk of dementia, and increases the risk of mortality from neurological disorders five-fold [4].

Hormone-replacement therapy (HRT) can reverse all these symptoms [4,5]; however, reluctance in prescribing HRT after hormone-dependent gynecological cancers is observed among physicians [2,6]. 

In this narrative review, we examine the recurrence risk associated with HRT use in menopausal women treated for OC, AC, and EC. The goal is to provide clarity on the potential scenarios in which HRT can be utilized, exercised with caution, or contraindicated, based on the best evidence from the literature.

### 1.1. Ovarian Cancer

In 2020, OC is estimated to be responsible for 314,000 new cases and 207,000 deaths worldwide [7]. It is the third most common gynecological cancer, after cervical and uterine cancer (age-standardized incidence for cervical, uterine, and ovarian cancer: 13.3, 8.7, and 6.6 per 100,000 females, respectively). Based on data from the U.S. National Cancer Database Surveillance, Epidemiology, and End Results (SEER), about 1.3 percent of women in the U.S. will be diagnosed with ovarian cancer at some point in their lives [8]. Incidence rates (per 100,000 population) vary by race/ethnicity.

Around 13.7% of OC patients are detected at a local stage and about 52% at a metastatic stage, where the 5-year survival rate is reduced to 29.7% instead of 91.8% if the diagnosis had been made before local spread. Ninety percent of ovarian tumors are epithelial, with the most common serous subtype [9].

The classification of OC includes epithelial and nonepithelial subtypes. More than 90% of ovarian malignancies are categorized as epithelial (EOC), and currently five main types have been identified: high-grade serous ovarian carcinoma (HGSOC) (70%), low-grade serous ovarian carcinoma (LGSOC) (<5%), mucinous (3%), endometrioid (10%), and clear-cell carcinoma (CCC) (10%) [10]. By now, these can be considered distinct diseases, as indicated by differences in epidemiological, molecular, and genetic factors. Nonepithelial ovarian carcinoma (NEOC) accounts for about 10% of all OC. The two most frequently diagnosed NEOCs are germ cell tumors (GCT) and sex cord-stromal cell tumors (SCST), and each of these classifications encompasses multiple histologic subtypes.

Surgical staging and cytoreduction, followed by adjuvant chemotherapy, is the management approach used for most patients with EOC. However, neoadjuvant chemotherapy (NACT) before definitive surgery is an alternative option in selected patients (e.g., stage IV disease, extensive extraperitoneal metastases, poor performance status) with biopsy-proven Müllerian malignancy. For patients whose disease is in remission after primary chemotherapy, maintenance therapy with poly(ADP-ribose) polymerase (PARP) inhibitors is offered. For patients with advanced OC and a germline or somatic mutation in BRCA1 or BRCA2 and a response to first-line platinum-based therapy, PARP maintenance inhibition is indicated [11].

Borderline ovarian tumors (BOT) are tumors with low-malignant potential that account for 10–20% of all epithelial tumors of the ovary [12]. They occur on average 10 years earlier and have a much better prognosis than OC (10-year survival of 97% for all stages combined [13]). The main histologic forms of BOT are serous and mucinous [14,15]. Current knowledge suggests that borderline serous tumors are part of a progression to low-grade serous cancer. Preoperative diagnosis of BOT requires precise preoperative examinations to differentiate benign from malignant tumors. Since the prognosis of BOT is commonly good and considering the young age of patients at onset, surgical treatment of patients with a desire for pregnancy is becoming increasingly conservative [12]. Fertility-sparing surgery is feasible for most women of reproductive age. Postoperative therapy is recommended for women with serous BOTs and invasive implants [16].

### 1.2. Cervical Adenocarcinoma

Cervical carcinoma (CC)represents the fourth most frequent cancer and the fourth leading cause of cancer death in the worldwide female population [7]. The estimated incidence of CC is 13 per 100,000 women, varying widely by geographic area, with rates ranging from less than two to seventy-five per one hundred thousand women when less-resourced countries are considered. This difference reflects the varying accessibility to screening programs, vaccination, and treatment that have greatly reduced the incidence and mortality of CC in industrialized countries [17]. The most frequent histologic type of CC is squamous cell carcinoma (SCC), which accounts for about 80%, while adenocarcinoma (AC) is less common, reaching about 20% [18].

CC is most frequently diagnosed in women aged 35 to 44 years, with a mean age at diagnosis of 50 years [19]. Treatment of CC varies according to the stage of the disease and is the same for both SCC and AC. In the early stages, the gold standard is radical surgery, consisting of pelvic lymphadenectomy, radical hysterectomy, and bilateral salpingo-oophorectomy. Ovarian preservation combined with an opportunistic salpingectomy may be considered, depending on age and risk factors. Possible ovarian transposition should be evaluated individually, taking into account risk factors for possible adjuvant radiotherapy. Treatment of CC in locally advanced stages is external beam radiotherapy (EBRT) with a dose of 45 Gy/25 fractions or 46 Gy/23 fractions (by use of intensity-modulated or volumetric arc technique) and concomitant chemotherapy (weekly cisplatin), followed by brachytherapy. Fertility-sparing treatment may be considered only in selected cases, after appropriate counseling, in patients with SCC and AC with tumor sizes < 2 cm who want to preserve the possibility of childbearing [18].

Papilloma virus (HPV) infection is universally recognized as the leading cause of CC, although other risk factors are known, including the use of hormone therapies [18]. In a major systematic review, it was reported that the relative risk of CC increases with increasing duration of therapy with combined oral contraceptives in HPV-positive patients [20]. Even though CC is not counted among hormone-dependent carcinomas, it has been shown that the cervix presents receptors for both estrogen and progesterone [20]; these receptors have also been demonstrated in HPV-related lesions and in CC, with high expression, especially in AC, in which the presence of hormone receptors is overexpressed in about one-third of cases [21,22]. The endocervix is a target tissue for circulating hormones as it responds to the hormone changes of the menstrual cycle, varying its mucus production. Due to its origin in the endocervical epithelium, which is more histologically similar to the endometrium, AC appears to share some risk factors with endometrial adenocarcinoma, which is a hormone-sensitive tumor [23].

Despite the high concentration of hormone receptors, no clear relationship between hormone stimulation and tumor proliferation in AC has been demonstrated, and the association between receptor status and oncological outcome has not been clarified. Therefore, no prognostic or clinical significance can be attributed to hormone receptor expression in AC-affected women [21].

### 1.3. Endometrial Cancer

EC is the sixth most diagnosed cancer in women, with 417,000 new cases and 97,000 deaths in 2020. Incidence rates vary widely between countries around the world, with the highest rates observed in North America, Europe, Micronesia/Polynesia, and Australia/New Zealand. EC is the most common gynecological cancer in Europe. Most of the patients are diagnosed at an early stage (80% in stage I), with a 5-year survival rate of more than 95% [7].

Endometrioid forms are related to hyperestrogenism, and the main risk factors are represented by nulliparity, late menopause, obesity, diabetes, hypertension, and estrogen therapy, which are not adequately counterbalanced by the progestogens. Estrogen-independent EC is instead associated with previous pelvic radiotherapy or previous use of tamoxifen.

The management of EC depends on several prognostic factors, and the definition of prognostic categories to lead the treatment has been updated to the 2020 ESGO/ESTRO/ESP guidelines, including molecular classification, which identifies four subgroups: POLE ultramutated, MSI hypermutated, copy number low, and copy number high [24]. The pivotal treatment is surgery with cytoreductive intent, making mini-invasive procedures the best option. The standard is simple hysterectomy with bilateral adnexectomy and pelvic and paraortic lymphadenectomy or evaluation of the sentinel lymph node, which, in specialized centers, has replaced the lymphadenectomy in early-stage disease.

Side-specific systematic lymphadenectomy should be performed in high-intermediate-risk/high-risk patients if a sentinel lymph node is not detected. In intermediate-high, high-risk, and advanced endometrial cancers, radiotherapy and chemotherapy play a basic role. Ovarian preservation can be considered in pre-menopausal patients aged <45 years with low-grade endometrioid endometrial carcinoma with myometrial invasion < 50% and no obvious ovarian or other extra-uterine disease; however, salpingectomy is recommended [25].

The treatment of EC in premenopausal women can lead to the induction of surgical menopause, and these women may experience vasomotor symptoms, bone mass loss, and vulvovaginal atrophy with sexual concerns [26].

## 2. Materials and Methods

From June 2023 to July 2023, we performed a narrative review through a literature search in PubMed, lead by the following research question: “How many articles discuss HRT as a recurrence risk in menopausal women treated for ovarian cancer, cervical cancer (adenocarcinoma), and endometrial cancer?”

The research methodology is detailed in Table 1.

After finding 56 results (22 for OC, 1 for AC, and 33 for EC), titles and abstracts have been screened independently by three review authors (V.E.B., M.D., and I.D.A.). Duplicates have been removed. Any disagreements between the parties were resolved through discussion with a fourth reviewer (P.V.). After titles and abstracts were carefully read, we selected a total of 47 articles that could meet our research question. We therefore applied some inclusion and exclusion criteria by reading the full texts (Table 2).

Each full text has been independently assessed by three authors (E.V.B., MD., and I.D.A.). We followed the same methodology for each query, selecting a total of 23 articles (11 clinical studies on OC, 5 clinical studies on AC, and 6 clinical studies on EC) that met our research question.

A search of the references of both potentially relevant articles and articles qualifying for inclusion was also performed; this method of inclusion is known as snowballing sampling [27], which consists of searching the main references provided in each article, and when some significant redundancies arose among the articles (e.g., flagship publications), we included them in our articles list (but not in the main tables, except for a retrospective review for EC, which was relevant for our research question).

Due to the nature of this review (narrative), a limitation can be placed on its selection process, based on the subjective sensitivity of the authors. Other articles not cited in this review may be valuable, as well as those that have been selected.

## 3. Results

### 3.1. HRT in Ovarian Cancer

Table 3 reviews current clinical studies assessing HRT after OC. Reviews and meta-analyses evaluated the effect of HRT after OC on survival, QoL, and disease recurrence, considering various factors. Clinical trials on HRT after OC are not numerous, although several reviews, meta-analyses, and a Cochrane study [28] have been published since 2010 to date, producing references for clinical practice.

In general, the results of these studies show no negative effect of HRT on the risk of recurrence. However, the data did not construct definitive results regarding new histopathologic findings or the characterization of OC.

Considering histological classification, although most meta-analyses do not highlight significant differences regarding histological subtypes of OC, most of them, as a precaution, report avoiding HRT in LGSOC [29,30] and granulosa cell tumors (GrCT) [30,31]. HGSOCs are not considered predominantly estrogen-dependent, so HRT might be administered [32,33,34,35,36]. In contrast, LGSOC has increased sensitivity to estrogen, and a recent evaluation considered HRT contraindicated [37]. Similarly, based on theoretical considerations, great caution should be exercised in endometrioid tumors with positive hormone expression [31,35]. For mucinous subtypes, HRT may be prescribed. Clear cell carcinoma (CCC) subtypes are associated with an increased risk of venous thromboembolism [38]; this could be worsened by a similar risk associated with systemic HRT. However, transdermal HRT might decrease this risk. Further evidence is needed to confirm this suggestion in patients with OC [39,40].

Only a few reviews have analyzed hormone receptor expression. The only clinical study reporting on hormone receptor assessment is that of Li et al. [41], in which log-rank analysis revealed no significant differences in cumulative survival time among patients with different expression of estrogen or progesterone receptors. Nevertheless, most of these reviews stated that caution should be exercised by women with hormone receptor expression. There is a positive effect of HRT on maintaining QoL, which overcomes the unreasonable doubt about the increased risk of recurrence [29,35,42,43]. Almost all studies showed that the type of HRT administered was composed of a high dose of estrogen with or without progestin (medroxyprogesterone acetate).

A recent statement by EMAS suggests that HRT could be offered to women with nonserous EOC and GCT, while caution is needed with both serous carcinoma and GrCT [44]. In particular, given the benefits observed with aromatase inhibitors in stage II-IV low-grade serous carcinoma [45], HRT is not recommended in these types. A similar conclusion, considering estrogen sensitivity, was drawn for advanced endometroid OC.

Among all the articles analyzed in the cited reviews, great variability was observed regarding the timing of initiation of HRT administration, ranging from 1 to 21 months after surgery [32,33,34,46]. The review by Angioli et al. specifically discussed the duration of HRT administration after OC in specific cases and suggested combined treatment for a short period, preferably <5 years [30]. In addition, the clinical study by Ji and colleagues [47] found that HRT administered for more than 5 years increased OS (HR = 0.234).

A recent Swedish clinical trial by von Kartaschew and colleagues studied HRT administration in 664 premenopausal women after OC [48]. Ninety-six patients had EOC, 61 had NEOC, and 207 had BOT. HRT dispensed to the total cohort ranged between 32% and 41% in the first 5 years after surgery. Most premenopausal women undergoing surgery did not use postoperative HRT, while those with BOT had a higher percentage of HRT dispensed than those treated for OC.

None of the published studies clearly demonstrated an adverse effect of HRT on OC patients. On the contrary, several studies and related meta-analyses showed that HRT does not increase OC recurrences or affect OS [43,49] and, in some cases, improves OS with longer treatment duration [47].

However, the lack of evidence for new histopathological findings induces caution about specific recommendations. Specifically, data on different histologic subtypes and molecular characterizations, such as the presence of hormone receptors or other genetic characterizations, are rare and inconsistent. Therefore, over the years, review findings have introduced pragmatic comments and suggested personalized counseling [30,31,50,51]. In most studies, the average duration of follow-up is low; therefore, another recommendation is to avoid long-term HRT supplementation.

Regarding BOT, current knowledge suggests its evolution into LGSOC, and Angioli et al. [30] recommend avoiding HRT. In addition, according to a recent systematic review [52], no data on hormone contraception after BOT was found. HRT showed a trend towards increased risk for serous BOT but no significant link with mucinous. Serous BOT with high-risk histological features, like micropapillary patterns, stromal microinvasion, or peritoneal implants, have a higher risk of hormone-sensitive recurrence. Therefore, using HRT after severe BOT with these high-risk factors requires careful consideration on an individual-case basis.

However, for women previously treated for mucinous and serous BOT without high-risk histological criteria, HRT can be prescribed without restrictions [52]. According to the French Guidelines regarding BOT management [53], following the treatment of mucinous BOT in women under 45 years old, considering the favorable effects of HRT on cardiovascular and bone health, and recognizing the hormone insensitivity of mucinous BOT, it is advisable to propose HRT. For women over 45 years of age, the prescription of HRT is contingent upon the presence of climacteric symptoms and should be determined after conducting an individual assessment of the benefits and risks.

**Table 3:** Studies on Ovarian Cancer recurrence after HRT.

Results	Case Type	Sample	Study Design	Endpoint	Reference
No significant difference in DFS (RR HRT 0.90; 95% CI 0.52–1.54) RR of dying in HRT patient was 0.73 (95% IC 0.44–1.20).	Serous: 26 Mucinous: 23 Endometrioid: 11 Adenocarcinoma: 14 Clear cell: 4	Case: 78 Control: 295	Cohort study	HRT use and OC OS and DFS	Eeles et al. (1991) [32]
The differences in DFI (*p* = 0.785) and OS (*p* = 0.354) between the two groups were not statistically significant.	Serous: 39 Mucinous: 16 Endometrioid: 2 Clear cell: 2	Case: 59 Control: 66	RCT	HRT use and OC OS and DFS	Guidozzi& Daponte (1999) [34]
The estimated risk of death with HRT was 0.90 (OR = 0.90; 95% CI 0.24–5.08).	Serous: 24	Case: 24 Control: 48	Cohort study	HRT use and OC OS	Uršič Vrščaj (2001) [33]
There was no overall difference in 5-year EOC survival according to use HRT before diagnosis. EOC survival HRT use (multivariate HR = 0.83, 95% CI = 0.65–1.08), except for serous EOC (HR 0.69, 95% CI 0.48–0.98). Better survival for EOC-patients who used HRT after diagnosis (multivariate HR 0.57, 95% CI 0.42–0.78). For women with BOT there were no associations between HRT-use pre- or postdiagnosis and survival.	Serous: 87 Mucinous: 16 Endometrioid: 42 BOT: 150 Other: 21	Case: 150 Control: 499	Cohort study	HRT use and OC OS	Mascarenhas et al. (2006) [54]
The SR did not differ significantly in patients with or without HRT (*p* > 0.05).	Serous: 21 Mucinous: 10	Case: 31 Control: 44	RCT	HRT use and OC OS rate	Li et al. (2012) [41]
HR for death in the HRT group compared with the control group was HR 0.67 (95% CI 0.18–2.50), and the HR for relapse was 0.72 (95% CI 0.39–1.35). No significant differences in survival or relapse between the two groups.	Serous: 28 Mucinous: 10 Endometrioid: 24 Clear cell: 12 Other: 3	Case: 77 Control: 77	Cohort study	HRT and OC OS and DFS	Wen et al. (2013) [42]
Ever vs. never use (HR 0.80, 95% CI 0.62–1.03) and a significant survival benefit in long-term HRT users (≥5 years use vs. never use, HR 0.70, 95% CI 0.50–0.99, *Ptrend*= 0.04)	Serous: 568 Mucinous: 74 Endometrioid: 126 Clear cell: 49 NOS: 164 Other: 44	HRT patients: ○ Case: 233 ○ Control: 299	Cohort study	HRT use and OC OS	Bešević et al. (2015) [49]
Long-term OS was superior in the HRT group (HR 0.63; 95% CI 0.44–0.90; *p* = 0.011). After adjustment for stratification factors the HR was 0.51 (95% CI 0.34–0.76).	Serous:29 Mucinous: 8 Endometrial: 11 Clear cell: 9 Undifferentiated: 7 Other: 11	Case: 75 Control: 75	Original report of a RCT	HRT use and OC OS and relapse-free survival	Eeles et al. (2015) [46]
HRT group and control group did not significantly differ in relapse rates, HR in HRT group: 0.290; 95%, CI 0.31–2.47)	Serous: 31	Case: 31 Control: 81	Cohort study	HRT use and OC OS and PFS	Zhang et al. (2016) [55]
In patients with <55 yrs, DFS is improved according to the multivariable landmark analysis (*n* = 68/145, adjusted HR 0.354, 95% CI 0.17–0.74, *p* = 0.006). No associations between HRT use and OS or DFS were found among women aged 55 years and older.	Endometrioid: 34 Clear cell: 19 Other: 41	Case: 94 Control: 263	Cohort study	HRT after non-serous epithelial OC association with a decrease in OS and DFS	Power et al. (2016) [56]
OS was significantly greater in the HRT group (HR 0.618; 95% CI 0.414–0.922; *p* = 0.018). OS was significantly higher in the HRT group (85.3% vs. 76.6%; *p* = 0.016). The ratio of women with HRT to women without HRT increased significantly with time (restricted mean survival times for OS, *p* < 0.001). OS was significantly greater for those that received HRT for >5 years than for those that received HRT for ≤0.5 years (HR 0.234; 95% CI 0.059–0.936; *p* = 0.040).	Mainly EOC	Case: 263 Control: 1521	Retrospective cohort	HRT use and OC OS	Ji et al. (2022) [47]

Abbreviations: BOT: borderline tumor; CI: confidence interval; DFS: disease-free survival; DFI: disease-free interval; EOC: epithelial ovarian cancer; HR: hazard ratio; HRT: hormone replacement therapy; NOS: not otherwise specified; OC: ovarian cancer; OR: odds ratio; OS: overall survival; PFR: progression-free survival; RR: risk ratio; RCT: randomized control trial; SR: survival rate;yrs: years.

### 3.2. HRT in Cervical Adenocarcinoma

Table 4 shows the studies in the literature on HRT in patients with AC. The scientific data that evaluates the effect of hormone therapy on the incidence of AC is scarce and inconsistent, highlighting a weak association between long-term hormone therapies and the incidence of AC rather than SCC.

In a multicenter case-control study, Lacey et al. aimed to establish the effect of hormone replacement therapy (HRT) on increasing the risk of CC for the first time, differentiating between SCC and AC [57]. A total of 124 women with AC, 139 women with SCC, and 307 healthy controls were enrolled in the study. Only thirteen cases of AC (10.5%), seven cases of SCC (5%), and twenty controls (6.5%) had used HRT. The use of HRT was associated with AC (OR = 2.1, 95% CI 0.95–4.6) but not with SCC (OR = 0.85, 95% CI 0.34–2.1); however, the association was statistically non-significant. No associations were observed with duration of use or age of first use of HRT and increased incidence of CC; only unoppressed estrogens demonstrated a positive trend to AC (OR = 2.7).

Another observational cohort study performed in Finland also confirms a positive association between AC and HRT use [58]. All women who performed HRT for at least 6 months (n = 243,857) at the age of 50 years were enrolled in the study. A total of 32 patients with SCC and 65 patients with AC were identified. Study results showed that HRT for less than 5 years was not associated with CC, while use for more than 5 years was associated with a reduced risk for SCC (standardized incidence ratios SIR = 0.34; 0.16–0.65) and with an increased risk for AC (SIR = 1.83; 1.24–2.59). The authors explain the increased risk of AC in HRT users for more than 5 years by the similarity between the endocervix and endometrium, both of which are sensitive to hormone action. Considering this confusing scientific evidence, the fear that hormone stimulation could promote the cancer cell’s proliferation has often limited the use of HRT in patients with CC, especially in patients with AC.

Treatment of CC, which often involves young patients, frequently causes early iatrogenic menopause, either due to radical surgery or chemo- and radiotherapy treatments that in most cases result in irreversible hypogonadism. Climacteric symptoms in these patients can be more severe than in physiologic menopause, significantly altering the quality of life. The sudden onset of estrogen deprivation, the young age, the psychological component, and the clinical complications associated with local treatments make climacteric symptoms more severe in these patients, especially those of the urogenital tract; in addition, early menopause increases the metabolic, cardiovascular, and osteoporotic risks related to premature hormone deprivation [29].

To the best of our knowledge, to date, there are no randomized clinical trials on the use of HRT in patients treated for CC. In addition, the data in the literature mainly focuses on HRT in SCC patients, while evidence on AC is even more scarce. The only randomized data analyzing the effect of HRT on patients treated for CC are those of Ploch in 1987. In this study, 120 patients with stage I and II CC were enrolled and followed for 5 years; 40 patients were treated with triphasic estrogen/gestagen preparations, 40 with sequentially dienestrol and chlormadinon, while the remaining 40 patients received no hormone treatment, representing the control group [59]. This study showed that HRT can control most climacteric symptoms as well as relieve post-radiological complications without interfering with cancer outcomes. In fact, the recurrence rate between the hormonally treated and the control group was not statistically significant (20% and 32%, respectively, *p* ≤ 0.05). Unfortunately, this study does not specify the histological type and does not differentiate the oncological outcome between SCC and AC.

A recent retrospective review evaluating the safety of HRT specifically in AC patients was published in 2021 [60]. In this review, all women with stage 1B–2B AC under the age of 50 were enrolled. A total of 58 women were divided into three groups: 25 (43.1%) patients with ovarian conservation, 20 (34.4%) patients with iatrogenic menopause treated with HRT, and 13 (22.4%) patients with untreated iatrogenic menopause. This study, although with a small case series, shows that HRT does not affect the survival of patients treated for AC; in fact, no statistically significant difference in overall, disease-specific, or progression-free survival was identified in the three groups.

Finally, Lee et al. [61] recently published a retrospective case-control study where the effect of tibolone treatment on the survival outcome of patients with AC was evaluated. A total of 70 patients surgically treated were enrolled: 38 treated with tibolone and 32 controls. Progression-free survival (*p* = 0.34) and overall survival (*p* = 0.22) were similar between the groups, as were the risks of progression and death, demonstrating that tibolone has no effect on the survival of AC patients and that it can be used safely in these women. 

**Table 4:** Studies on Cervical Adenocarcinoma recurrence and HRT.

Results	Case Type	Sample	Study Design	Endpoint	Reference
HRT enabled control of most climacteric symptoms 5-year OS HRT+ vs. HRT−: 80% vs. 65% Recurrence rate HRT+ vs. HRT−: 16 (20%) vs. 13 (32%)	All CC.	Patients after surgery or RT for stages I and II CC: 120 ○ Treated with triphasic estrogen/gestational preparations (*n* = 40). ○ Treated with sequentially dienestrol and chlormadinone (*n* = 40). ○ Control group (*n* = 40).	Prospective randomized	To evaluate the effect of HRT on QoL and on oncological treatment	Ploch (1987) [59]
HRT− AC OR: 2.1 HRT− SCC OR: 0.85	SCC and AC	Women with CC (*n* = 263). Control group (*n* = 370).	Multicenter case-control	To evaluate the risk of developing CC in patients using HRT	Lacey et al. (2000) [57]
Any use of HRT ○ SCC SIR: 0.41 ○ AC SIR: 1.31 <5 years of HRT ○ SCC SIR: 0.66 ○ AC SIR: 0.73 >5 years of HRT ○ SCC SIR: 0.34 AC SIR: 1.83	Precancerous lesions, SCC, and AC	Women receiving HRT (*n* = 243,857) Control group: Finnish cancer statistics.	Cohort study (observational).	To evaluate the use of HRT and risk of precancerous lesions, SCC, and AC.	Jaakkola et al. (2012) [58]
PFS (*p* = 0.34) and OS (*p* = 0.22) were similar in both groups The risks of progression (HR 1.71; 95% C, 0.46–6.37; *p* = 0.43) and death (HR 1.59; 95% CI 0.06–45.66; *p* = 0.79) were also similar	AC	70 AC patients with FIGO stages IA to IB Tibolone: Users (*n* = 38) Non-users (*n* = 32)	Retrospective case-control	To evaluate the effects of tibolone on the survival of AC patients	Lee et al. (2018) [61]
No statistically significant difference in OS, disease-specific survival (73% in IM-NOHRT, 95% in IM-HRT, and 95% in OVCON), or PFS (68% in IM-NOHRT, 90% in IM-HRT, and 81% in OVCON)	AC	58 women with stage 1B-2B AC ○ *n* = 25 (43.1%) had ovaries conserved (OVCON) ○ *n* = 20 (34.4%) had IM-HRT ○ *n* = 13 (22.4%) had had IM-NOHRT	Retrospective review	To evaluate if HRT in patients treated for AC is detrimental to survival	Richardson et al. (2021) [60]

Abbreviations: AC: adenocarcinoma; CC: cervical cancer; IMHRT: iatrogenic menopause with HRT; IM-NOHRT: iatrogenic menopause without HRT; OS: overall survival; OVCON: ovary conserved; PFS: progression-free survival; SCC: squamous cell carcinoma; SIR: standardized incidence ratio; QoL: quality of life.

### 3.3. HRT in Endometrial Cancer

In women who underwent surgery for EC, the studies published over the past decades do not support an increased recurrence rate after the initiation of HRT [62]. Several small observational studies have consistently found that recurrence rate and disease-free survival (DFS) were not worse in women treated for stage I-II EC; only in one study patients with stage III disease were included (Table 5).

The first study conducted in 1986 by Creasman et al. analyzed 221 patients with stage I EC, 47 (21%) of whom were treated with oral and/or vaginal estrogen with a dosage of 0.625 or 1.25 mg. The other 174 patients were compared with a control group. The HRT was started with a median interval of 15 months after cancer treatment, and the estrogen group had a statistically significant longer DFS [63]. In 1990, Lee and coworkers examined 143 patients treated for stage I EC, 44 of whom were selected for HRT with 0.625 or 1.25 mg of oral equine conjugated estrogens (CEE), and 99 were selected as a control group. Fifty-seven percent of the patients started HRT within 12 months after surgery. No significant differences were found in the recurrence rate between the two groups, as there were no recurrences in the estrogen group while there were eight in the control group [64]. In the same year, two small studies were published by Baker and Bryant on 31 and 20 women taking HRT after EC, respectively, and showed no recurrences in these cancer survivors [65,66].

Chapman et al. published a retrospective analysis of 123 patients who had surgery for stage I and II EC in 1996. No significant differences in DFS were found between the HRT group and the non-estrogen group (*p* = 0.163). Ninety-two percent of HRT patients had taken CEE with a mean dose of 0.64 mg/day, and most had started it within 12 months after surgery; 33 women received, in combination with estrogen, an average dose of 2.5 mg/day of progesterone [67].

Suriano and coworkers are the only ones to have included patients treated for stage III EC. Among the 75 women who received HRT, most started taking it within 6 months after surgery and used a daily dose of 0.625 mg of CEE, and 49% additionally took 2.5 mg of medroxyprogesterone acetate (MPA). A comparison between the HRT group and the control group showed a significantly longer DFI for the former. Furthermore, this study shows a protective effect of pretreatment HRT exposure [68].

The study published by Ayhan et al. in 2006 shows, like others, that the use of HRT in EC survivors does not increase the rate of recurrence or death rates. The 50 patients analyzed started HRT with 0.625 mg CEE plus 2.5 mg MPA 4–8 weeks after surgery [69]. The only prospective randomized controlled trial reports a risk of recurrence within 36 months in the estrogen (CEE) versus placebo group of 1.27 (80% CI 0.916–1.77) and an absolute recurrence rate of 2.1% [70]. In a sub-analysis, the use of estrogen alone significantly increased the risk of tumor recurrence when used in black women (HR 11.2, 95% CI 2.9–43.3) but not in white women (HR 1.24, 95% CI 0.17–8.80) [71]. A meta-analysis of nearly 900 HRT patients vs. 1100 controls even showed that estrogen plus progestin HRT had a protective effect against cancer recurrence (OR 0.23; 95% CI 0.08–0.66), whereas estrogen-only therapy did not show this effect (OR 0.35, 95% CI 0.06–2.10). Most of the studies included in the meta-analysis used the standard dosage of 0.625 mg of oral CEE; only some administered a daily dose of 1.25 mg [72].

In the most recent systematic review, DFS and disease recurrence analysis confirm that HRT did not increase the risk of EC recurrence (HR 0.90, 95% CI 0.28 to 2.87; and OR 0.63, 95% CI 0.48 to 0.83). Also, in the subgroup analysis by tumor stage, hormone therapy type, timing, and duration, only analyzing data by ethnicity, Black American women were found to be at an increased risk of EC recurrence (HR 7.58, 95% CI 1.96 to 29.31). The most risk-reducing HRT was estro-progestin (OR 0.63, 95% CI 0.47–0.84) and the cyclic regimen with 0.625 or 1.25 mg CEE (OR 0.12, 95% CI 0.02 to 0.64), and considering the tumor stage, it was most effective in FIGO I (OR 0.12, 95% CI 0.02–0.64). Considering the estrogen-only regimen studies where the vaginal route was used, both OR and HR were slightly more protective than in studies where only an oral administration was considered [73].

**Table 5:** Studies on Endometrial Cancer recurrence after HRT.

Results	Case Type	Sample	Study Design	Endpoint	Reference
Time to recurrence (*p*< 0.05), with the estrogen group experiencing longer disease-free survival.	Stage I EC	Cases (*n* = 47) Controls (*n* = 174)	Retrospective cohort	To evaluate why HRT was controindicated in patients treated for EC.	Creasman et al. (1986) [63]
No significant difference in recurrence rate was found between low-risk estrogen and low-risk nonestrogen users.	Low-risk (early-stage, low grade) EC	Cases (*n* = 44) Controls (*n* = 99)	Retrospective case-control	To evaluate the use of HRT in low-risk EC patients.	Lee et al. (1990) [64]
No significant differences in DFS were found between the HRT group and the non-estrogen group.	Stage I and II EC	Cases (*n* = 62) Controls (*n* = 61)	Retrospective case-control	To evaluate the use of HRT in early-stage EC and the risk of recurrence or death.	Chapman et al. (1996) [67]
Longer disease-free interval among the hormone replacement group (*p* = 0.006).	Stage I, II, and III EC	Cases (*n* = 75) Controls (*n* = 75)	Retrospective cohort	To evaluate if HRT increases the risk of recurrence or death in EC patients.	Suriano et al. (2001) [68]
No increased risk of recurrence or death.	Stage I and II EC	Cases (*n* = 50) Controls (*n* = 52)	Prospective case-control	To evaluate oncologic outcome of HRT in EC patients.	Ayhan et al. (2006) [69]
Tumour recurrence 2.3% in HRT arm versus 1.9% in the placebo arm.	Stage I and II EC	Cases (*n* = 618) Controls (*n* = 618)	RCT	To evaluate the effect of HRT on recurrence rate and survival in stage I and II EC.	Barakat et al. (2006) [70]
Black patients in the ERT group had a significantly increased risk of disease recurrence compared with those in placebo group (*p* = 0.012).	Stage I and II EC	Black patients (*n* = 110) White patient (*n* = 1049)	Retrospective review (using GOG 137 trial participants)	To evaluate racial disparity between black and white patients with early-stage EC.	Maxwell et al. (2008) [71]

Abbreviations: DFS: disease-free survival; EC: endometrial cancer; GOG: gynecological oncology group; HRT: hormone replacement therapy; RCT: randomized controlled trial; ERT: estrogen-only replacement therapy.

## 4. Discussion

Treatment of hormone-dependent gynecological cancers can have a negative impact on the QoL of young women forced into iatrogenic menopause, which can cause hot flushes, symptoms of GSM, bone loss, and cognitive impairment [1]. Bone mass can decrease by as much as 3% a year in the first 5 years after menopause [74], and this percentage can be higher in women with cancer treatment-induced bone loss (CITBL) [75].

**Figure 1:**
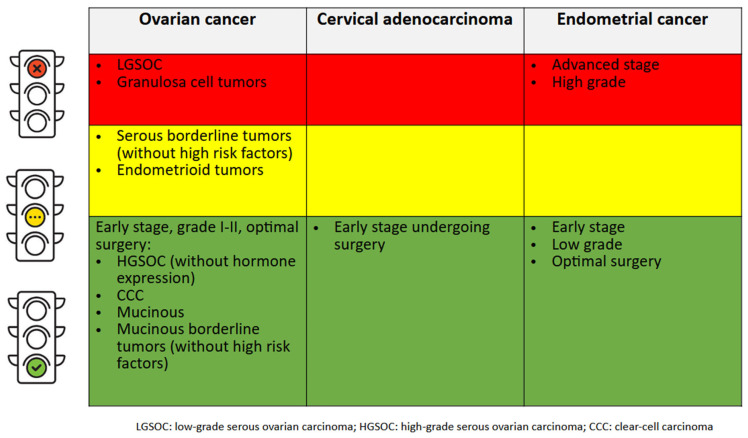
Clinical suggestions for a short course of HRT for women who experience troublesome menopausal symptoms that cannot be relieved by alternative non-hormone therapy after hormone-dependent gynecological cancer. LGSOC: low-grade serous ovarian carcinomas; HGSOC: high-grade serous carcinoma; CCC: clear cell carcinoma.

HRT can help reverse all these symptoms [4,5], but it is not clear if it can be safely prescribed after OC, AC, and EC since they are hormone-dependent gynecological cancers. The results of the findings of this review are summarized in Figure 1.

### 4.1. Ovarian Cancer

Women treated for OC who experience debilitating menopausal symptoms and worsening QoL should be followed by experienced gynecologists, who can evaluate the advantages and disadvantages of HRT in this population. Any future elaboration of recommendations must consider several factors, such as histological subtypes, hormone receptor status, and tumor stage. Currently, some general considerations can be highlighted to help clinicians, based on the literature of low quantity and medium quality, but considering the constant updating of preclinical research:

- HRT can be introduced after the end of treatment.
- HRT should not be prescribed concurrently with biologic or experimental treatments for OC.
- Postmenopausal HRT should be prescribed only to women who experience debilitating menopausal symptoms that cannot be relieved by alternative non-hormone therapy.
- HRT should possibly be prescribed to women with early stage, grade I–II OC, and those who have undergone optimal surgery.

These considerations may refer to a short course of HRT (2–5 years). In cases of early iatrogenic menopause, a multidisciplinary approach should be implemented to decide whether HRT should be continued for a longer period. Further consideration can be applied to BOT:

- Serous borderline tumors: caution should be exercised in administering HRT, as current knowledge suggests that borderline serous tumors are part of a progression to low-grade serous tumors, the latter being recognized as a hormonally responsive tumor.
- Mucinous borderline tumors without risk factors: HRT may be administered.

Prescription HRT based on endocrine receptors should be considered preferentially in HGSOC without hormone expression, in mucinous, and in CCC. It is less indicated in endometrioid, and not recommended in LGSOC.

### 4.2. Cervical Adenocarcinoma

Our review highlighted that for AC, there is currently no data demonstrating an effect of HRT on recurrence rate or cancer outcome. Similarly, there is no evidence to suggest the superiority of one treatment to another. HRT varies depending on the treatment of CC. Surgically treated patients with radical hysterectomy, are proposed an estrogen-only therapy. For patients undergoing radio-chemotherapy, on the other hand, continuous combined therapy is proposed. Considering the limited scientific evidence, it is clear that HRT should be carefully evaluated on a case-by-case basis and after appropriate counseling in patients with AC. The considerations we can draw are that:

- HRT is not contraindicated in AC survivors.
- Treatment should be prescribed by gynecologic oncologists experienced in the management of these patients.
- The regimen of therapy (unopposed or opposed estrogen) depends on the presence or absence of the uterus.
- Therapy can be prescribed both to patients at an early stage undergoing surgery and to patients with locally advanced stages who are receiving exclusive radio-chemotherapy.

Further prospective randomized data are needed to definitively define and clarify the safety and efficacy of HRT in AC survivors.

### 4.3. Endometrial Cancer

Finally, there is no evidence in vivo that after surgery, estrogens stimulate the growth of residual cancer cells, and EC metastatic cells have reduced expression of hormone receptors, so it is possible that disease progression depends on alternative oncogenic pathways [76]. Studies and guidelines indicate that HRT is a possibility to treat menopausal symptoms and prevent the long-term consequences of hypoestrogenism in women surviving EC, although they are based on low-quality evidence [5,62,73,77,78]. The indications we can draw are the following:

- HRT can be prescribed to treat uncomfortable symptoms of menopause in women with early-stage low-grade EC after surgery.
- There are not enough data to give indications in women treated for more advanced FIGO stages EC. Therefore, HRT is not advised with high-grade, advanced-stage endometrial carcinoma.
- From the available data, no definite conclusions can be reached about the hormone type, mode, or duration.

Because the analysis of estrogen alone and estroprogestin has been done in different types of studies, it remains uncertain whether HRT after EC should contain progestin or not, but in the general population of postmenopausal women, the risks of combined HRT exceed those of estrogen alone [73]. Regarding the estrogen-only regimen, vaginal estrogen administration has not been studied separately, but considering that it does not appear to be associated with an increased risk of recurrence and the impact of progestins on the breast, it appears to be a valid therapeutic option [5]. Any treatment choice must be made by weighting the risks and benefits with the patient and individually evaluating each case.

### 4.4. Strengths and Limits 

Our review provides a comprehensive outline of the existing literature, and several limitations must be acknowledged. Firstly, the included studies vary in their sample size as well as their quality, resulting in a heterogeneous database. This variability could influence the consistency and reliability of the findings. Additionally, a notable limitation is the lack of long-term data on the safety and efficacy of HRT after gynecologic cancer. Finally, it is worth noting that the types of HRT evaluated in most of the studies differ from those currently available on the market. Historically, HRT regimes were characterized by higher dosage regimens. However, contemporary formulations feature lower dosage regimens, incorporate new-generation progestins, and offer a broader range of application forms. Thus, it would be reasonable to anticipate favorable outcomes with these newer formulations and to plan further research to address this knowledge gap. 

## 5. Conclusions

In conclusion, advances in the early detection and treatment of gynecologic malignancies have improved patient survival. However, these gains are often accompanied by a variety of treatment-associated toxicities that negatively influence QoL. 

For women with hormone-dependent gynecologic cancer undergoing optimal surgery and experiencing significant vasomotor symptoms unresponsive to nonhormonal strategies, HRT can be prescribed by experienced gynecologists who can weigh the pros and cons in this population. HRT can be introduced after the end of adjuvant treatment, and it should not be prescribed concurrently with biological or experimental treatments. Further prospective randomized data are needed to definitively define and clarify the hormone type, mode, or duration of HRT in this subset of patients.

## Figures and Tables

**Table 1:** PubMed search methodology.

Total	Language	Article Type	Query [Title/Abstract]
22	English	Clinical trial Meta-analysis RCT	“ovarian cancer” AND (“HRT” OR “Hormone Replacement Therapy”)
1			(“cervical adenocarcinoma” OR “cervical cancer”) AND (“HRT” OR “Hormone Replacement Therapy”)
33			“endometrial cancer” AND (“HRT” OR “Hormone Replacement Therapy”)

**Table 2:** Inclusion and exclusion criteria.

Exclusion Criteria	Inclusion Criteria
Impact of surgical treatments on patients Non-anti-estrogen therapies Counselling of disease career Risk factors Non-steroidal anti-inflammatory therapies Advanced stage disease Second malignancy Non appropriate surgical-pathologic staging procedures	HRT after treatment for OC Only post cancer Only cancer (not endometriosis) Type of OC: ○ Invasive epithelial ○ Borderline ○ Rare histotypes Type of CC: ○ Cervical adenocarcinoma Type of EC: ○ Endometrial adenocarcinoma
